# Antimicrobial oral lavage reduces the SARS-CoV-2 load in intubated patients: randomized clinical trial

**DOI:** 10.1080/20002297.2022.2152179

**Published:** 2022-12-13

**Authors:** Letícia Mello Bezinelli, Luciana Corrêa, Stephany Beyerstedt, Érika Bevilaqua Rangel, Carlos Benitez, Nelson Hamerschlak, João Renato Rebello Pinho, Debora Heller, Fernanda de Paula Eduardo

**Affiliations:** a Hospital Israelita Albert Einstein, São Paulo, Brazil; b School of Dentistry, University of São Paulo, São Paulo, Brazil; c Nephorology Division, Federal University of Sao Paulo, São Paulo, Brazil; d Latin American Oral Health Association, São Paulo, Brazil; e Postgraduate Program in Dentistry, Universidade Cruzeiro do Sul, São Paulo, Brazil; f Department of Periodontology, School of Dentistry, The University of Texas Health Science Center at San Antonio, San Antonio, Texas, USA

**Keywords:** SARS-CoV-2, COVID-19, Orotracheal intubation, Oral fluid, Chlorhexidine, Hydrogen peroxide

## Abstract

**Background:**

The oral cavity can be a reservoir for SARS-CoV-2 and may play a crucial role in the viral transmission in the hospital environment.

**Objective:**

To investigate whether an oral hygiene protocol with chlorhexidine (CHX) used alone and in combination with hydrogen peroxide (HP) in the intensive care unit was effective in reducing the SARS-CoV-2 viral load in the oral cavity.

**Methods:**

SARS-CoV-2 viral load was measured on oral fluid samples collected from patients undergoing orotracheal intubation. The study sample was randomly in: CHX group (n = 19) - oral rinse using only 0.12% CHX solution; HP+CHX group (n = 24) - oral rinse with 1.5% HP and 0.12% CHX. The samples were collected before the interventions (T0), immediately (T1), 30 minutes (T2) and 60 minutes (T3) after the procedure.

**Results:**

A significant viral load reduction was observed at T1 (mean ± SD:–0.57 ± 0.19 log10;–73.2%;p = 0.022) in the HP+CHX group. No statistically significant differences between any time points were observed in the CHX group.

**Conclusion:**

The HP+CHX oral rinses significantly reduced the SARS-CoV-2 viral load in the oral fluid immediately after the procedure. The CHX oral rinse alone did not result in any significant viral load reductions.

## Introduction

The global COVID-19 pandemic has given rise to a critical need for high-level multidisciplinary medical care in hospitals where frequent transmission of the SARS-CoV-2 virus between patients and healthcare workers may occur, particularly during administration of aerosol-generating treatment procedures [1,2]. In this context, the role of the oral cavity in the transmission of COVID-19 must be considered, with previous evidence suggesting high prevalence of SARS-CoV-2 and elevated viral titers in the saliva of patients diagnosed with COVID-19 [3–5]. Therefore, reduction of the viral load in the patient’s oral cavity and upper respiratory tract is crucial for minimizing the risk of nosocomial infections among patients and health care providers.

Orotracheal intubation is associated with an increased risk of SARS-CoV-2 transmission due to the close proximity of the health care professional to the patient’s airway and the likelihood of aerosol production during administration of treatment [6]. Additionally, it may also result in an increased risk of ventilator-associated pneumonia (VAP) in patients diagnosed with COVID-19 [7] due to the aspiration of oral bacteria which may play a crucial role in the pathogenesis of VAP. The adoption of certain oral hygiene protocols such as decontamination of the mouth using a non-alcoholic solution of chlorhexidine gluconate (CHX) with or without tooth-brushing [8] in intensive care units (ICUs) may help reduce the risk of VAP. On the other hand, currently there is limited evidence on the efficacy of this intervention in reducing SARS-CoV-2 viral load.

Previous *in vitro* [9–12] and clinical studies [13–15] examining the effects of various CHX solutions on viral load have reported contradictory results. Recently, our research group performed a pilot clinical trial to test the efficacy of various oral rinses, including CHX alone or in combination with hydrogen peroxide (HP+CHX), in reducing SARS-CoV-2 viral load in the saliva of patients diagnosed with mild to moderate COVID-19 and hospitalized in negative pressure rooms [16]. We found that the HP+CHX solution exhibited promising results. HP is a potent antiviral solution used for surface decontamination [17], and has been recognized as an effective oral debriding agent with antiseptic properties when used at a concentration of 1.5% (.

The aim of the current study was to investigate if the CHX oral hygiene protocol used for VAP prevention, combined or not with HP, could reduce the SARS-CoV-2 viral load in the oral fluid of patients diagnosed with COVID-19 and undergoing orotracheal intubation in the ICU.

## Methods

### Institutional review board approval

The current study was approved by the Ethics Committee on Human Research, Hospital Israelita Albert Einstein, Brazil (project no. 32,018,820.5.0000.0071), and is in accordance with The Code of Ethics of the World Medical Association (Declaration of Helsinki). The study was also registered at www.trial.gov (no. NCT04537962). Informed consent for participation in the study was collected from the patient’s family members/legal guardians as the patients themselves were unconscious.

### Trial design

This study was a prospective, randomized, double-blinded, single-center clinical trial design with two parallel groups to assess the equivalence of the two groups for the reduction in SARS-CoV-2 viral load.

### Participants

This study included patients diagnosed with COVID-19 and admitted into the ICU at Hospital Israelita Albert Einstein (HIAE) between May and July 2020. Male and female patients who were aged ≥18 years; exhibited SARS-CoV-2 positivity (detected by RT-PCR test using an oropharyngeal sample); were undergoing orotracheal intubation for ≤4 days at the time of sample collection; and demonstrated no contraindications for the use of CHX and HP were included in this study. Patients who tested negative for SARS-CoV-2 using an oropharyngeal sample; did not require orotracheal intubation or underwent intubation for >4 days; were <18 years of age; exhibited presence of ulcerations and/or other oral injuries that contraindicated the topical use of CHX and HP; presented with oral bleeding that prevented sample collection; reported side effects associated with topical use of CHX and HP; or were unwilling to cooperate during the intervention and collection of oral fluid samples were excluded from this study.

### Interventions

The interventions were performed by two senior dentists previously trained in the administration of oral care in intubated patients. The patients were divided into two groups based on the interventions used, as follows: a) CHX group- oral rinse using 5 mL of non-alcoholic 0.12% CHX gluconate solution for 1 min (Periogard®, Colgate-Palmolive Company); and b) HP+CHX group – oral rinse with 5 mL of 1.5% hydrogen peroxide solution for 1 min (Peroxyl®, Colgate-Palmolive Company) followed by 5 mL of non-alcoholic 0.12% CHX gluconate solution for 1 min (Periogard®). The solutions were administered by gently rubbing a cotton swab on the ventral and dorsal surfaces of the tongue, upper and lower lip mucosae, buccal mucosa, hard and soft palate, and gingiva. During the interventions, a high-powered suction was used to prevent the spreading of the solution. A rigid biosecurity protocol was followed by the healthcare professionals during all interventions and sample collections in order to prevent SARS-CoV-2 contamination.

### Primary outcome

The primary outcome for this study was the reduction (in percentage) in SARS-CoV-2 viral load in the oral fluid samples collected after intervention and measured using quantitative reverse transcription PCR (RT-qPCR).

### Sample size estimation

The sample size was estimated based on the SARS-CoV-2 load observed in the samples collected from 13 patients in each group. Power calculation taking the estimated variability, detection of a minimum difference of 1 viral log reduction, 80% power, 95% confidence interval, and 5% alpha into consideration showed that the CHX and HP+CHX groups required 18 and 23 patients, respectively. Therefore, the current study included 25 and 29 patients in the CHX and HP+CHX groups, respectively, in order to compensate for any potential dropouts during the course of the study.

### Randomization and blinding

Two senior researchers were responsible for randomly allocating patients to the intervention groups using computer-generated codes. The dentists were not blinded as one of the interventions required administration of two sequential oral rinses. The allocation concealment was kept throughout the study to the laboratory staff responsible for administration of the RT-qPCR tests as well as to the statisticians analyzing the data.

### Oral fluid collection

The senior dentists collected oral fluid samples prior to the intervention (T0) as well as immediately (T1), 30 minutes (T2), and 60 minutes (T3) after the procedure by gently scraping a cotton swab on the dorsal surface of the tongue, upper and lower lip mucosae, and floor of the mouth. The samples were then placed in a sterile saline solution (total volume 3 mL), stored on ice, and transported to the laboratory for RNA extraction after T3. The interval from the first collection at T0 to RNA extraction was 1h30min.

### Total RNA extraction and quantitative reverse transcription PCR analysis

RNA was extracted from the oral swab sample using the QIASymphony DSP Virus/Pathogen kit (QiagenHilden, Germany) as per the manufacturer’s instructions. SARS-CoV-2 was detected using the RT-qPCR method (XGEN Master COVID-19; Mobius Life Science, Pinhais, Brazil). Briefly, 5 μL of extracted RNA was added to 15 μL of a mixture of enzymes, probes, oligonucleotides, buffer, and deoxynucleotide triphosphates (MIX CV19, XGEN Master COVID-19), and the viral nucleocapsid (N), viral polyprotein ORF1ab, and human RNase P genes were amplified. Positive controls were used during RNA extraction (presented in the MEGAscript T7 Transcription kit, Thermo Fisher Scientific) and during amplification (presented in the XGEN Master COVID-19 kit). Viral RNA extracted from SARS-COV-2 virus culture supernatant used for standard curve quantification was also a positive control. This curve was established by serial dilutions from 2 × 10^1^ to 2 × 10^6^ copies/mL. The SARS-CoV-2 supernatant culture was obtained through nasopharyngeal samples from COVID-19-positive Brazilian patients [18], which has been used for validating COVID-19 diagnostic tools in Brazil [19]. Negative controls (nuclease free water-only sample previously used for RNA elution) were added in each RT-PCR run. Thermal cycling was performed using the QuantStudio™ Real-time PCR System (ThermoFisher Scientific, Waltham, MA, USA), and consisted of reverse transcription at 45°C for 15 minutes, followed by 95°C for 2 minutes, 45 cycles of 95°C for 10 seconds each, and 60°C for 50 seconds. The tests were triplicated, and cases with a cycle threshold value of >40 were considered as SARS-CoV-2 undetectable. Viral load was established in copies/mL.

### Clinical data

Clinical data including age, gender, race, risk factors, comorbidities, the duration of ventilation, total length of ICU and hospital stay, and extension of lung lesions were recorded.

### Statistical analysis

Numerical data were reported as mean, standard deviation (SD), median, and minimum/maximum values, while categorical data were presented in absolute and relative (%) frequencies. Chi-squared test or Student’s t test were used to compare baseline clinical data between the groups. The SARS-CoV-2 viral load was logarithmically transformed (base10) prior to statistical analysis assessing differences between time intervals (T0 vs. T1, T0 vs. T2, and T0 vs. T3) for each treatment group. Within-treatment comparisons of the SARS-CoV-2 viral load (log_10_) in the baseline and follow-up samples were performed using a generalized estimating equation (GEE) model, and post-GEE pairwise comparisons of the study treatments were performed using the Bonferroni’s test for multiple comparisons. Two-sided statistical tests at a significance level of 0.05 were carried out using the statistical software, SPSS (IBM Corp.).

## Results

### Participant flow and recruitment

**Figure 1.**
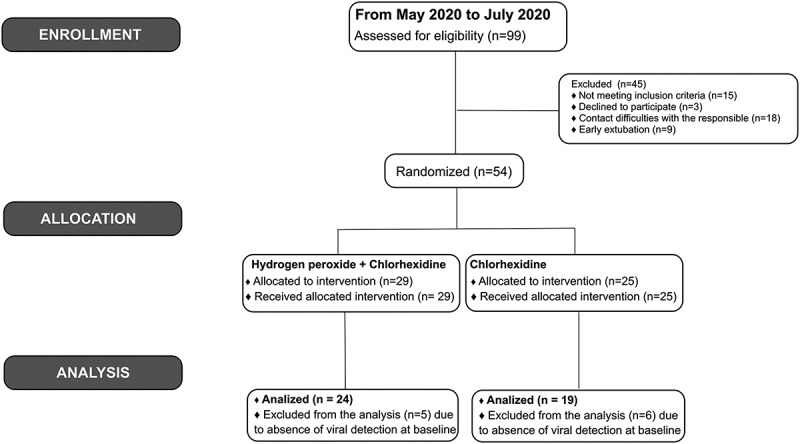
Flow chart showing selection of final study cohorts from eligible patients admitted into the COVID-19 intensive care unit between May and July 2020.

Between May and July 2020, 99 patients diagnosed with COVID-19 and admitted into the HIAE ICU were assessed for inclusion eligibility and 45 were excluded from this study. After randomization of the remaining 54 patients, 11 patients exhibited no virus at T0 and were excluded. The final CHX and HP+CHX intervention groups consisted of 19 and 24 patients, respectively (Figure 1).

### Baseline data

Table 1 shows the demographic and clinical data of the study sample at baseline. The majority of patients were male in both groups. The CHX group had a median age of 62 years old and duration of ICU stay of two days, while the corresponding values in the HP+CHX groups were 59 years old and three days of ICU stay.

**Table 1.** Clinical data* of ICU patients included in the current study.

Variable	Chlorhexidine group (n = 19)	Hydrogen peroxide + Chlorhexidine group (n = 24)	P value
Male – n (%)	13 (68.4)	15 (62.5)	0.685
Female – n (%)	6 (31.6)	9 (37.5)	
Age – median (range)	62 (30–82)	59 (24–87)	0.737
Days in ICU – median (range)	2 (1–4)	3 (1–4)	0.243
Days of oro-tracheal intubation – median (range)	1 (1–4)	2 (1–4)	0.027
Patients with comorbidities – n (%)	10 (52.6)	14 (54.2)	0.708
Diabetes	4 (21.1)	5 (20.8)	
Renal disease	2 (10.5)	2 (8.3)	
Obesity	1 (5.3)	1 (4.2)	
Cardiovascular disease	2 (10.5)	6 (25.0)	
Smoking	4 (21.1)	1 (4.2)	
Respiratory disease	3 (15.8)	4 (16.7)	
Hypertension	5 (26.3)	7 (29.1)	
Oncologic patient	0 (0.0)	3 (12.5)	
Hypothyroidism	1 (5.3)	1 (5.2)	
Parkinson disease	0 (0.0)	1 (4.2)	
Extension of lung lesion – n (%)			
25–49%	5 (26.3)	10 (41.7)	0.294
50%	2 (10.5)	2 (8.3)	0.805
>50%	7 (36.8)	12 (50.0)	0.388

* Data obtained at the time of first oral fluid sample collection. P value for chi-squared test or Student’s t test.

The median duration of oro-tracheal intubation was one day in the CHX group and two days in the HP+CHX group, and this difference was statistically significant (p = 0.027). The frequency of comorbidities was similar between the two groups, with lung lesion extensions being the most commonly observed comorbidity (>50%).

### Outcomes

**Figure 2.**
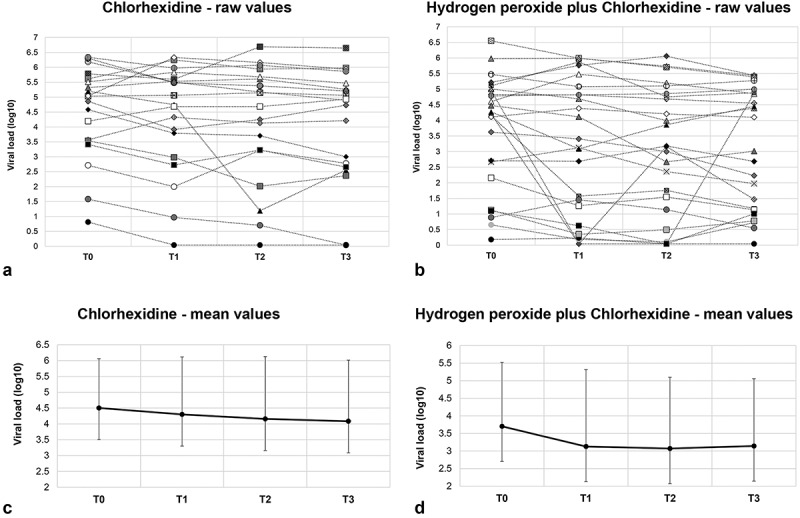
Individual (A and B) and mean ± standard deviation (C and D) values of SARS-CoV-2 viral load (log_10_) detected in oral fluid samples using the RT-qPCR method. The samples were collected from intubated patients who underwent oral rinses using either 0.12% chlorhexidine solution (CHX group) or 1.5% hydrogen peroxide followed by 0.12% chlorhexidine (HP+CHX group). The oral fluid samples were collected before (T0) the intervention and immediately (T1), 30 minutes (T2), and 60 minutes (T3) after the oral rinse procedure.

The individual viral load (log_10_) values and mean ± SD values for the CHX and HP+CHX groups have been shown in Figure 2.

**Figure 3.**
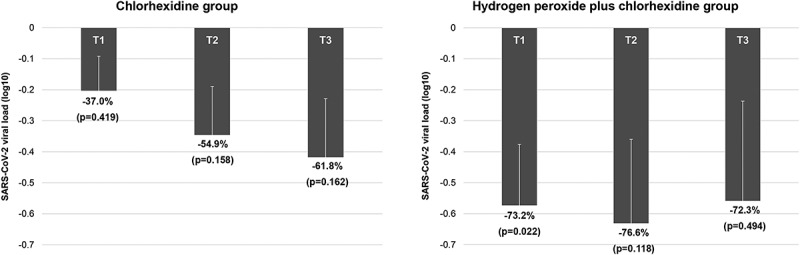
Mean ± SD values of SARS-CoV-2 viral load reduction (log_10_) in patients treated with oral rinses using A) 0.12% chlorhexidine or (B) 1.5% hydrogen peroxide followed by 0.12% chlorhexidine. The viral load values observed immediately (T1), 30 minutes (T2), and 60 minutes (T3) after the intervention were compared to those seen at baseline (T0). Percentage viral load reduction has been shown, and the p-values were calculated using generalized estimating equation (GEE) models.

Both groups exhibited a trend toward reduction of viral load from baseline (T0) to 60 minutes after the interventions (T3) (Figure 3). In the HP+CHX group, a significant difference between T0 and T1 was observed (p = 0.022), and the mean (± SD) reduction at this time point was −0.57 (± 0.19 log_10_; −73.2%). This group also exhibited the highest viral load reduction (−0.63 log_10_; −76.6%) at T2, but this difference was not statistically significant. The CHX group exhibited no statistically significant differences between baseline and the follow-up time points (Figure 3).

## Discussion

To the best of our knowledge, this is the first study to examine the effects of antimicrobial oral rinses on SARS-CoV-2 viral load in the oral cavity of patients undergoing orotracheal intubation. The aim of the current study was to investigate whether inclusion of HP in the previously established CHX-based oral hygiene protocol in intubated patients would result in a greater reduction of SARS-CoV-2 viral load in oral fluid samples. The alternate hypothesis was confirmed and a significant reduction in viral load immediately after the intervention (T1) was observed in the HP+CHX group. However, this effect was transitory and no statistically significant differences were observed between the other time points.

Although HP has a significant oxidative effect that can disrupt the viral envelope and degrade the viral RNA [20], it is possible that the low substantivity of the HP solution limited its antiviral effect to a short time period. Based on its long-term antibacterial effect, we had previously hypothesized that CHX would maintain its antiviral effect [21]. The mean viral load reduction was seen to persist 30 and 60 minutes after the intervention in the HP+CHX group; however, the log values exhibited greater variance in the oral fluid samples over time and this could be related to the SARS-CoV-2 viral load dynamics in the patient’s airway and saliva. Nevertheless, the mean reduction in viral load consistently remained above 50% at all time points, suggesting that oral rinses using a combination of HP and CHX in intubated patients were effective in minimizing the viral load of SARS-CoV-2.

CHX alone was also seen to cause a reduction in SARS-CoV-2 viral load in the oral fluid samples over time, although the differences between baseline and the various follow-up time points were not statistically significant. This result prevents the formation of any definitive conclusions regarding the effectiveness of 0.125% CHX against SARS-CoV-2. Previous studies examining the efficacy of CHX solutions on SARS-CoV-2 viral load have reported inconsistent results, with one *in vitro* study observing high efficacy of 0.2% CHX solution in the inactivation of SARS-CoV-2 [11] and others concluding that CHX solutions at concentrations of 0.1% and 0.2% had limited effect against the virus [9,10,12]. Although the efficacy of 0.12% CHX oral rinses and oropharyngeal sprays and mouthwash in the reduction of SARS-CoV-2 viral load have been previously demonstrated in two clinical trials [4,14,15], another clinical study with fewer patients have reported a transient antiviral effect [15]. None of these studies included COVID-19-positive ICU patients undergoing orotracheal intubation.

Contraindications of the HP+CHX solutions need to be clearly established. Our group does not use these solutions when the oral mucosa is atrophic and/or exhibits ulcerated or hemorrhagic lesions due to the negative effect of these solutions on tissue repair, especially of CHX on oral mucosa cells. In addition, the presence of ulcerated or hemorrhagic surfaces can improve the effervescence derived from HP-induced oxygen release, which can be difficult to control in the ICU and can improve the aerosol effect. Therefore, we believe that the combination of HP and CHX could be applied only for ICU patients with severe COVID-19 but without oral lesions.

The key limitation of this study was the absence of a placebo group as this would allow comparison of viral load reduction with and without oral rinses using antimicrobial solutions. However, as the interventions were primarily performed for the prevention of VAP, the increased risk of nosocomial infections in the COVID-19 ICU ward prevented us from including a placebo group. Another limitation was the fact that we did not measure the isolated HP effect on SARS-CoV-2 load because all the patients had a high risk of ventilator-associated pneumonia, and we followed the institutional oral hygiene protocol with chlorhexidine. Therefore, the effect of HP alone needs to be further investigated The current study used CHX at a concentration of 0.12%; concentrations of 0.2% and even 2% [8] could potentially have greater antiviral efficacy. Further studies examining the effects of higher concentrations of CHX on SARS-CoV-2 viral load in ICU patients are necessary.

## Conclusion

A combination of HP and CHX oral rinses significantly reduced the SARS-CoV-2 viral load in the oral fluids of intubated patients immediately after the intervention, while CHX oral rinses alone were not associated with any significant reductions in viral load.

## Clinical trial number

XXX
